# GAS5 long non-coding RNA in malignant pleural mesothelioma

**DOI:** 10.1186/1476-4598-13-119

**Published:** 2014-05-23

**Authors:** Arun Renganathan, Jelena Kresoja-Rakic, Nohemy Echeverry, Gabriela Ziltener, Bart Vrugt, Isabelle Opitz, Rolf A Stahel, Emanuela Felley-Bosco

**Affiliations:** 1 Laboratory of Molecular Oncology, Clinic of Oncology, University Hospital Zürich, Zürich, Switzerland; 2 Division of Thoracic Surgery, University Hospital Zürich, Zürich, Switzerland; 3 Institute of Surgical Pathology, University Hospital Zürich, Zürich, Switzerland

**Keywords:** Malignant pleural mesothelioma, Long non-coding RNA, RNA FISH, Quiescence, Cell cycle length

## Abstract

**Background:**

Malignant pleural mesothelioma (MPM) is an aggressive cancer with short overall survival. Long non-coding RNAs (lncRNA) are a class of RNAs more than 200 nucleotides long that do not code for protein and are part of the 90% of the human genome that is transcribed. Earlier experimental studies in mice showed GAS5 (growth arrest specific transcript 5) gene deletion in asbestos driven mesothelioma. GAS5 encodes for a lncRNA whose function is not well known, but it has been shown to act as glucocorticoid receptor decoy and microRNA “sponge”. Our aim was to investigate the possible role of the GAS5 in the growth of MPM.

**Methods:**

Primary MPM cultures grown in serum-free condition in 3% oxygen or MPM cell lines grown in serum-containing medium were used to investigate the modulation of GAS5 by growth arrest after inhibition of Hedgehog or PI3K/mTOR signalling. Cell cycle length was determined by EdU incorporation assay in doxycycline inducible short hairpinGAS5 clones generated from ZL55SPT cells. Gene expression was quantified by quantitative PCR. To investigate the *GAS5* promoter, a 0.77 kb sequence was inserted into a pGL3 reporter vector and luciferase activity was determined after transfection into MPM cells. Localization of GAS5 lncRNA was identified by *in situ* hybridization. To characterize cells expressing GAS5, expression of podoplanin and Ki-67 was assessed by immunohistochemistry.

**Results:**

GAS5 expression was lower in MPM cell lines compared to normal mesothelial cells. GAS5 was upregulated upon growth arrest induced by inhibition of Hedgehog and PI3K/mTOR signalling in *in vitro* MPM models. The increase in GAS5 lncRNA was accompanied by increased promoter activity. Silencing of GAS5 increased the expression of glucocorticoid responsive genes glucocorticoid inducible leucine-zipper and serum/glucocorticoid-regulated kinase-1 and shortened the length of the cell cycle. Drug induced growth arrest was associated with GAS5 accumulation in the nuclei. GAS5 was abundant in tumoral quiescent cells and it was correlated to podoplanin expression.

**Conclusions:**

The observations that GAS5 levels modify cell proliferation *in vitro,* and that GAS5 expression in MPM tissue is associated with cell quiescence and podoplanin expression support a role of GAS5 in MPM biology.

## Background

Malignant pleural mesothelioma (MPM) are tumors originating from the surface serosal cells of the pleura [1]. MPM are rare tumors mainly caused by exposure to asbestos and patients have a median survival around 12 months even after combined chemotherapy [2,3]. Molecular studies identified altered expression of critical genes in oncogenesis, especially tumor suppressor genes at the INK4 and NF2 loci (reviewed in [4]). A recent study has shown dysregulation of long non-coding RNA (lncRNA) expression in MPM compared to normal mesothelium [5]. LncRNA are part of the transcriptome that does not encode for proteins, which includes tens of thousands of lncRNA [6]. LncRNAs are defined as being longer than 200 nucleotides [7] and have poor sequence conservation across species [8] which led to the hypothesis that the function is most likely linked to the RNA structure itself. The functions of some lncRNAs have been described and include chromatin modifier, transcriptional and post-transcriptional regulator of gene expression [9-11]. In this context, lncRNAs are now emerging as mammalian transcription key regulators in response to developmental or environmental signals [12-14] and are associated to many cancer related pathways through gene regulation [15]. Although much has still to be investigated about the major part of lncRNAs, the involvement of some of them in tumor progression has already been shown, e.g. lncRNA HOTAIR which interacts with the chromatin-remodelling complex PRC2 [16].

Interestingly, a minimal region of deletion was identified in asbestos induced murine malignant mesothelioma which includes *gas5* locus [17]. The *GAS5* gene is a so-called host gene for small nucleolar RNA (snoRNA) and it is encoded at locus 1q25. It has up to 12 exons and 10 box C/D snoRNAs within its alternative introns together with conserved 5′-terminal oligopyrimidine tract (5′ TOP) [18]. GAS5 is named based on the finding that its expression levels increased upon cell growth arrest induced after serum starvation [19] or as the result of rapamycin-induced cell cycle arrest [18]. Recent studies have shown that GAS5 silencing in T cells increased the proportion of cells in S phase, reduced the rate of spontaneous apoptosis [20] and protected cells from rapalogue (temsirolimus, everolimus) induced proliferation arrest [21]. In epithelial cells GAS5 regulates glucocorticoid-dependent transcription by acting as a decoy outcompeting the DNA-binding site of the glucocorticoid receptor, thereby reducing cell metabolism [22]. More recently GAS5 has been described to act as sponge which sequesters miR-21 [23]. GAS5 is also part of lncRNA abundantly expressed in cancer cells [24]. In this study, we investigate whether GAS5 has a role in MPM biology.

## Results

### GAS5 lncRNA expression level is lower in MPM cell lines compared to normal mesothelial cells and it is increased by drugs inducing growth arrest

**Figure 1:**
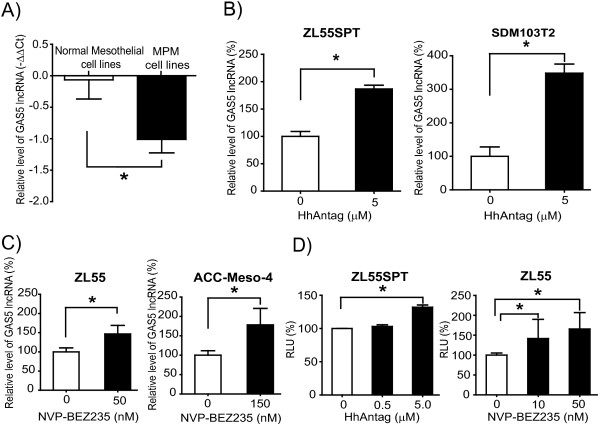
**GAS5 expression is lower in MPM and it is increased upon drug induced growth arrest. A**. GAS5 is lower in MPM cell lines compared to normal mesothelial cell lines. GAS5 lncRNA expression was analysed by qRT-PCR in 22 MPM cell lines and in 7 normal mesothelial cell lines. Expression of GAS5 was normalized to internal control histones relative to the mean expression of GAS5 in normal mesothelial cells in culture according to –∆∆Ct method. **B** and **C**. Drug induced growth arrest increases GAS5 expression in MPM primary cells (ZL55SPT and SDM103T2) and cell lines (ZL55 and ACC-Meso4). **D**. Expression of GAS5 promoter reporter gene was analysed in control versus HhAntag and NVP-BEZ235 treated samples. Promoter activity, expressed as relative light unit (RLU) normalized to control set at 100%, is significantly increased by treatment with HhAntag and NVP-BEZ235. Values are expressed as mean ± SD from three independent experiments *; p < 0.05.

GAS5 expression in MPM cell lines (n = 22) is significantly lower (Figure 1A; p < 0.005; individual MPM cell line profile is shown Additional file 1: Figure S1) when compared to normal mesothelial cells (n = 7). The *GAS5* gene produces 29 different splice variants (Additional file 2: Table S1) including 10 processed lncRNAs containing or not one or more snoRNA sequences, and 19 unprocessed sequences. The only information available on splice variants expressed in experimental models is provided by one study, where the expression of both mature and unprocessed GAS5 was observed in phytohaemagglutinin stimulated primary lymphocytes [20]. In order to investigate which splice variant is expressed in MPM, we used two sets of primers, theoretically allowing the detection of 20 different cDNAs of GAS5 splice variants (Additional file 3: Table S2) to amplify GAS5 cDNA from four mesothelioma cell lines (ZL55SPT, SDM103T2, ZL55 and ACC-Meso4) that had been selected for functional studies. According to the size of PCR fragments observed for each primer set (Additional file 4: Figure S2) and taking into account the detection by the two primer sets, the list of cDNA expressed was narrowed down to three splice variants including two lncRNA and one intron retaining transcript (Table 1). The expression of the intron retaining transcript was further confirmed using specific primers (Additional file 4: Figure S2D). Quantitative abundance of the different transcripts varied depending on the cell line (Additional file 4: Figure S2B, C and D). To investigate whether GAS5 expression in MPM cells could be modulated by drugs inducing growth arrest, we treated MPM cells with either HhAntag or with NVP-BEZ235 as previously described [25,26]. ZL55SPT and SDM103T2 cells, grown in serum-free medium and at 3% of oxygen conditions, which allow maintenance of dedifferentiation properties [25], where treated during 48 h with HhAntag. A significant (p < 0.05) increase of GAS5 lncRNA levels (Figure 1B) and of the number of quiescent, ki67 negative (G_0_) cells (Additional file 5: Figure S3) was observed compared to control. Similar results were observed by treating ZL55 and ACC-Meso4 cells, which are grown in serum-containing medium, during 6 h with GI50 (Echeverry, ms in preparation) concentration of NVP-BEZ235 (Figure 1C). While it is not possible to investigate the effect of HhAntag in cell grown in serum containing medium [25], a significant increase of GAS5 levels was observed after treatment of ZL55SPT and SDM103T2 to NVP-BEZ235 (Additional file 6: Figure S4), indicating that cells grown in dedifferentiating conditions remain sensitive to this drug.

**Table 1:** List of GAS5 alternative splice variants corresponding to the size of RT-PCR products

	**Vega Genome Browser 54 Transcript ID**	**Length (bp)**	**Type**
**GAS5-Ex-4-8**	**207 bps**		
	**OTTHUMT00000090577**	632	LncRNA
	OTTHUMT00000090578	1698	Retained Intron
	OTTHUMT00000090585	632	Retained Intron
	**OTTHUMT00000090586**	712	Retained Intron
	OTTHUMT00000090590	688	LncRNA
	OTTHUMT00000090593	772	Retained Intron
	OTTHUMT00000090598	979	Retained Intron
	**168 bps**		
	**OTTHUMT00000090579**	565	LncRNA
	OTTHUMT00000090595	745	Retained Intron
	OTTHUMT00000090597	497	Retained Intron
	OTTHUMT00000090605	723	Retained Intron
**GAS5-Ex-6-12**	**281 bps**		
	**OTTHUMT00000090577**	632	LncRNA
	**OTTHUMT00000090586**	712	Retained Intron
	OTTHUMT00000090590	688	LncRNA
	**242 bps**		
	**OTTHUMT00000090579**	565	LncRNA
	OTTHUMT00000090584	542	LncRNA
	OTTHUMT00000090604	413	LncRNA

Transcripts that were detected by both primer sets are in bold.

To investigate whether increased GAS5 RNA levels observed after treatment with HhAntag and NVP-BEZ235 were associated with increased GAS5 promoter activity, we transfected ZL55SPT and ZL55 cells with a luciferase reporter gene under the control of GAS5 promoter sequences (pGL3-B-pGAS5). Luciferase activity was measured 24 hours after growth arrest-inducing treatment. Although basal pGL3-B-pGAS5 was very high (approximately a thousand fold higher than empty vector, data not shown), a dose dependent significant (p < 0.05) increase in promoter activity was identified in ZL55SPT and ZL55 cells after HhAntag and NVP-BEZ-235 treatment (Figure 1D), respectively. Altogether, these results suggest that GAS5 may participate to the growth inhibitory action of HhAntag and NVP-BEZ235 in MPM cells.

### Silencing of GAS5 in MPM cells increases glucocorticoid receptor responsive genes and shortens the cell cycle length

**Figure 2:**
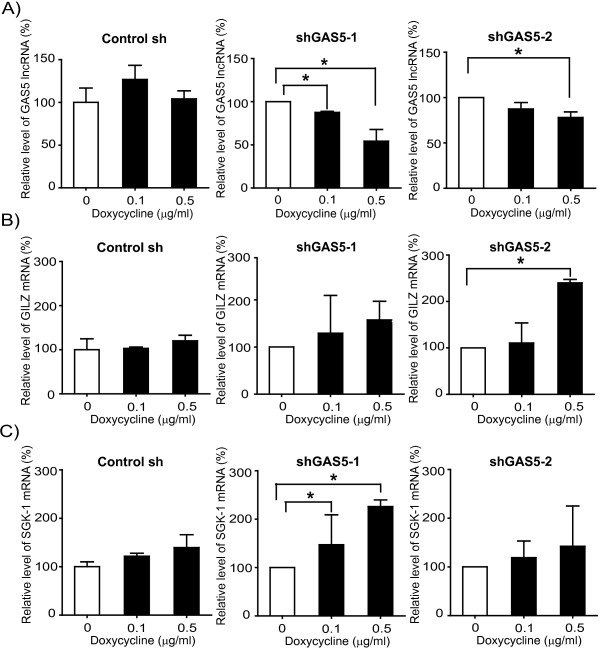
**Silencing of GAS5 increases glucocorticoid responsive genes. A**. GAS5 lncRNA silencing increases the expression of glucocorticoid responsive genes GILZ **(B)** and SGK1 **(C)**. Values are normalized with histones and shown as mean ± SD from three to four independent experiments. *; p < 0.05.

To investigate whether GAS5 plays a role in MPM growth, stable doxycycline-inducible shRNA-GAS5 clones were generated using ZL55SPT cells. GAS5 expression levels decreased in a doxycycline dose-dependent manner in shGAS5-1 and shGAS5-2 while it was not affected in control cells (Figure 2A). The partial resistance to siRNA-mediated knockdown is consistent with what has been recently described [27] for nuclear lncRNA (see below). To determine whether the observed 35 and 25% (in shGAS-1 and shGAS5-2, respectively) decrease in GAS5 expression was functionally relevant, we determined the consequences on glucocorticoid regulated genes such as glucocorticoid inducible leucine-zipper (GILZ) and serum/glucocorticoid-regulated kinase-1 (SGK1). Indeed, GAS5 lncRNA functions as a decoy for glucocorticoid receptor [22]. As ZL55SPT cells are grown in a serum-free medium containing pharmacological concentrations of glucocorticoids, which are essential for optimal cell growth [25,28], we expected that modulation of GAS5 levels would modify the expression of glucocorticoid regulated genes. Accordingly, we observed that doxycycline-induced decrease of GAS5 expression in shGAS5 clones was accompanied by a significant increase of expression of GILZ in shGAS5-2 clone (Figure 2B) and of SGK1 in shGAS5-1 clone (Figure 2C) while no change was observed in sh control clone, indicating a negative role of GAS5 in the expression of these genes. These observations were confirmed using three independent GAS5 siRNA. Indeed, we observed a significant down-regulation of GAS5 accompanied by a significant increase of GILZ mRNA for the three siRNA tested compared to a control siRNA and a significant increase of SGK1 mRNA for two of the three tested siRNA (Additional file 7: Figure S5).

**Figure 3:**
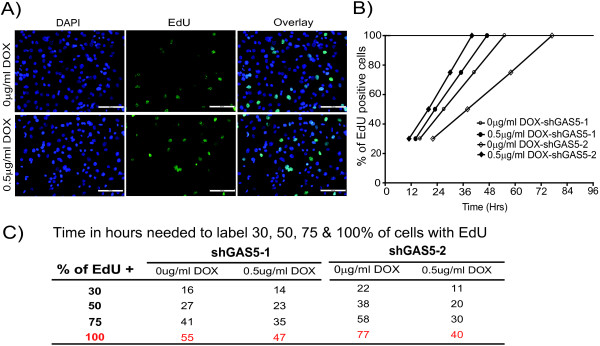
**Silencing of GAS5 shortens the cell cycle length as determined by EdU incorporation assay. A**. Representative micrographs of shGAS5 cells pre-treated with or without doxycycline during 72 h then incubated with EdU for 12 hrs. Scale bar: 100 μm. **B**. Cell cycle length was measured from a time course of EdU incorporation. Five different fields were imaged for each group in each of three independent experiments under fluorescence microscope and number of EdU positive cells was counted in approximately 1200 cells (DAPI). The graph plots the percentage of EdU positive cells vs. time in axes. The time taken for 100% of EdU positive cells was determined from the linear regression and slopes in presence or absence of doxycycline were compared p < 0.0001. **C**. Time in hours needed for 30, 50, 75 and 100% of cells to become EdU positive calculated according to the linear regression method.

Next we determined whether GAS5 knockdown affects cell cycle. In order to measure cell cycle length, we performed a time course experiment after pulse labelling shGAS5-1 and shGAS-2 cells with 5-ethynyl-2′-deoxyuridine (EdU). EdU assay revealed an increase in EdU positive cells after doxycycline-induced silencing of GAS5 in shGAS5 clones (Figure 3A and B) while no effect was observed in control cells (data not shown). Using a linear regression method [29], we determined the time taken for 100% EdU incorporation, which corresponds to the time needed for single cell cycle length (Figure 3B). The cell cycle length of uninduced shGAS5 clones was 55–77 hours while the GAS5 silenced cells exhibited a shorter cell cycle of 40 to 47 hours (Figure 3C). Collectively these results confirm that silencing GAS5 has functional consequences in the growth of MPM cells grown in serum-free conditions.

### HhAntag-induced growth arrest is accompanied by GAS5 accumulation into the nuclei

**Figure 4:**
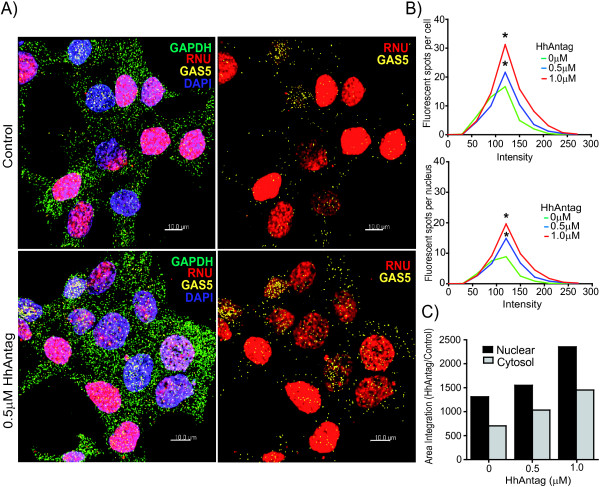
**Nuclear enrichment of GAS5 lncRNA after induction of growth arrest by HhAntag. A**. Left panel: Representative micrographs of fluorescence *in situ* hybridization showing GAS5 (yellow), GAPDH (green) for cytosolic control, RNU (red) for nuclear mRNA control, and nuclei stained with DAPI (blue). Right panel: same image as left panel showing only RNU and GAS5. Scale bar: 10 μm.** B**. Quantification of GAS5 in ZL55SPT cells and nuclei was determined by calculating intensity of fluorescent spots by Imaris image analysis using spot detection method. The graph plots the fluorescent spots per cell vs. intensity. *; p < 0.05 compared to the non-treated control. **C**. Integration of area under the curve to evaluate the proportion of nuclear GAS5 in control and HhAntag-induced growth arrest. Representative of three independent experiments.

To further characterize the mechanism of GAS5 in controlling cell growth in serum-free conditions, we analysed the subcellular localization of GAS5 after treatment with HhAntag by *in situ* hybridization. We confirmed a dose-dependent significant (p < 0.05) increase in GAS5 accumulation in the cells and observed an enrichment into the nucleus (Figure 4A and B). Quantification revealed that GAS5 is twice as abundant in the nucleus as in the cytosol in the absence of HhAntag and prevalence in the nucleus was maintained after treatment with HhAntag (Figure 4C). Altogether, these data indicate that HhAntag-induced growth arrest involves accumulation of nuclear GAS5.

### Podoplanin expression correlates with GAS5 in malignant pleural mesothelioma

**Figure 5:**
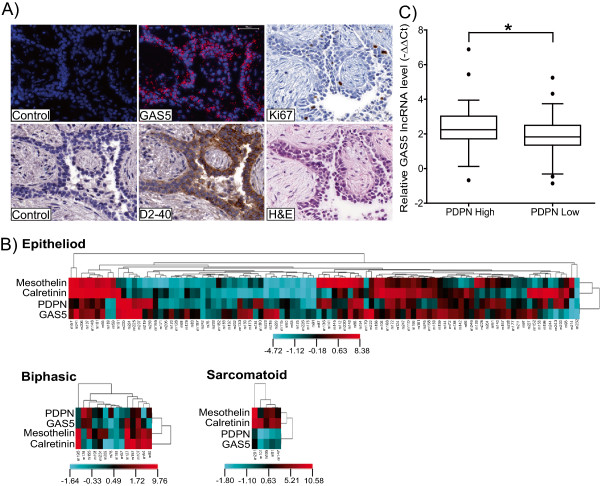
**GAS5 expression is higher in podoplanin positive MPM tumor cells. A**. Fluorescence *in situ* hybridization and immunohistochemical analysis of GAS5, Ki-67 and podoplanin in a representative patient. Upper panel shows fluorescence *in situ* hybridization of control and GAS5, immunostaining of Ki-67 (ab15580, Abcam). Lower panel shows the immunostaining of control and podoplanin (D2-40, DAKOCytomation) and hematoxylin and eosin stained tumor tissue. Scale bar: 50 μm. **B**. Matrix of relative gene expression in epitheliod, biphasic and sarcomatoid mesothelioma shown as heat map. Heat map is a grid of rectangles with colors that indicate the value of the matrix elements, where high expression is blue and low expression is red. Rows of each heat map correspond to genes, whilst columns correspond to MPM tumor samples. **C**. GAS5 expression is higher in MPM tissue expressing higher levels of podoplanin *p < 0.05, Mann–Whitney test.

In order to determine the relevance of our *in vitro* findings in clinical samples we determined GAS5 relative expression in MPM and normal tissue. Surprisingly, we found a significant six-fold (p < 0.0001) increase of GAS5 level in tumor tissue (n = 116) when compared to non-tumoral samples (n = 10) (Additional file 8: Figure S6A). This was unexpected considering the upregulation GAS5 during growth arrest and the shortening of cell-cycle observed upon GAS5 silencing. Because MPM tissue contains stromal cells [30] including immune system cells that are highly enriched for GAS5 expression [21], we performed *in situ* hybridization in a subset of samples to investigate and characterize the cells expressing GAS5. GAS5 expression was predominantly identified in quiescent tumor cells (Figure 5A). To further characterize GAS5 expression in relationship to MPM tumors we therefore investigated the relationship of GAS5 expression with mesothelial markers such as mesothelin, calretinin and podoplanin extending a previous analysis of these markers performed on a limited set of samples [30]. Hierarchical clustering of these mesothelial markers with GAS5 showed that the expression of GAS5 clustered with podoplanin in epithelioid, biphasic and sarcomatoid tissue samples (Figure 5B) and a positive correlation between GAS5 and podoplanin levels (p < 0.05) was observed (Figure 5C). Furthermore, GAS5 expression was higher in samples with high podoplanin (D2-40) expression (Figure 5A and Additional file 8: Figure S6B). No correlation was observed between GAS5 and mesothelin or calretinin.

Overall, these results indicate that *in vivo* GAS5 expression may play a role not only in growth control but may have additional functions such as the already described miRNA sponge activity [23].

## Discussion

In this study we revealed the functional consequences of the expression of GAS5, a lncRNA abundantly expressed in MPM. When compared to normal mesothelial cells, MPM cell lines expressed lower levels of GAS5 and exposure to drugs inducing growth arrest increased GAS5 expression level, in line with what had been previously described with rapamycin-induced cell cycle arrest of NIH3T3 cells [18]. We identified that increased GAS5 levels were associated to increased promoter activity. This is important since it has been shown that GAS5, as other mRNAs carrying 5’ TOP, can be stabilized by interaction with La motif-related protein 1 [31]. Interestingly, basal levels of GAS5 promoter were quite high. The promoter is likely to be bidirectional (Additional file 9: Figure S7) controlling both *GAS5* and *zinc finger and BTB domain containing 37* (*ZBTB37*). Not much is known about *ZBTB37* except that it is a transcription factor known to be expressed during embryonic development [32]. It might be interesting to verify whether *ZBTB37* is expressed in MPM after that the mechanisms of induction of *GAS5* promoter have been elucidated.

GAS5 plays an essential role in growth arrest state of both T-cell lines and non-transformed lymphocytes [21]. Silencing of GAS5 resulted in shortening cell cycle in MPM cells demonstrating that also in these cells GAS5 controls cell growth. The underlying mechanism can be linked to the fact that GAS5 is a glucocorticoid receptor-decoy and it inhibits transcription of glucocorticoid responsive genes [22]. Glucocorticoids are essential for optimal MPM cell growth [25,28] and are present at pharmacological concentrations in culture medium [33,34]. We estimated that GAS5 is present at the level of ten thousand copies/cell, which is in the range of the reported abundancy of glucocorticoid receptors [35-37]. Therefore it is not surprising that under our experimental conditions a change of glucocorticoid-regulated genes was observed upon GAS5 silencing. Quantitative *in situ* hybridization allowed determining that two thirds of GAS5 is present in the nucleus. Assuming that 10% of total cellular volume is occupied by nucleus this means that nuclear GAS5 concentration is approximately 18 fold higher in the nuclei compared to the cytosol. It had already been shown that GAS5 is located both in cytoplasm and the nucleus and translocates from the cytoplasm into the nucleus with glucocorticoid receptor in response to dexamethasone [22] but quantitative data were not available. Because there are cell to cell variations in the nuclear abundancy of GAS5 and 35% of cells are in quiescent state, it is tempting to speculate that cells with high nuclear levels of GAS5 are quiescent cells. Although we could not address this question in cultured cells, since *in situ* hybridization of nuclear RNA is not compatible with e.g. Ki-67 immunohistochemistry within the same specimen, we observed in MPM tumors that GAS5 expressing tumor cells were Ki-67 negative supporting the idea of nuclear GAS5 being associated with growth arrest. Besides interference with glucocorticoids signalling, other mechanisms may involve chromatin remodelling, since GAS5 expression is increased after growth arrest induced by silencing of Brahma ATPase subunit of mammalian SWI/SNF complexes [29]. The latter are a family of chromatin remodeling enzymes that regulate gene expression by disrupting histone-DNA contacts in an ATP-dependent manner.

After drug treatment inducing growth arrest GAS5 levels were higher. Interestingly, genome-wide data on RNA and protein quantities in proliferating vs. quiescent yeast cells has revealed that although the proteome size is similar in proliferating and quiescent cells, the transcriptome is decreased to 20% in quiescent compared to proliferating cells [38], indicating that the increase in GAS5 levels observed upon drug treatment inducing cell-cycle arrest are probably underestimated in the context of whole transcriptome.

GAS5 was mostly accumulating in the nucleus after growth arrest induced by HhAntag, suggesting that the control of cell cycle is dependent on nuclear GAS5. Control of cell cycle mediated by lncRNA has been demonstrated for MALAT1, whereby silencing MALAT1 resulted in decreased proliferation because MALAT1 is required for mitotic progression [39] during which MALAT1 migrated from the nucleus to the cytoplasm shuttling heterogeneous ribonucleoprotein C, which is necessary to increase IRES-dependent translation of *c-myc*[40]. Altogether, these data support that nuclear-cytoplasmic shuttling of lncRNA is tightly regulated by mechanism controlling cell cycle.

Surprisingly, GAS5 was expressed at higher level in tumor tissue compared to non-tumoral tissue. This in contradiction with other tumor tissues such as breast [41], bladder [42] and pancreatic [43] cancer where GAS5 expression was lower in tumor compared to non-tumoral tissue. One reason might be that primers recognizing all GAS5 ESTs were used in the breast cancer study [41], while in our case primers were recognizing only 20 out of 29 different splice variants, which nevertheless included the splice variants detected in cancer cells. In the other two studies primer sequence was not specified so the comparison is not possible. Another explanation for this discrepancy could have been that GAS5 expression seems tissue-dependent, accordingly to publicly available integrated database on transcriptome [44]. Therefore, a high expression in tumoral tissue could have resulted from e.g. lymphocytes infiltration. By *in situ* hybridization of MPM tissue we observed a high GAS5 expression in quiescent tumor cells. In order to find an explanation why GAS5 expression should be high in tumor cells, we compared GAS5 expression to known mesothelioma markers and found that GAS5 expression was positively correlated to podoplanin expression. The latter is a type I transmembrane sialomucin-like glycoprotein, which induces platelet aggregation and which is highly expressed in malignant pleural mesothelioma [45]. Due to the coincidence of GAS5 expression with podoplanin immunoreactivity and the fact that GAS5 has been recently demonstrated to have miRNA sponge activity [23] one possibility is that high GAS5 expression is not only controlling cell cycle, but it is acting as miRNA natural sponge [46] to scavenge podoplanin mRNA degradation by miRNA, which is known to occur at least in glioblastoma [47]. Further work is necessary to investigate this hypothesis, nevertheless sequence analysis revealed shared miRNA target sequence in GAS5 and podoplanin (http://www.mircode.org).

## Conclusions

In conclusion, accumulating evidences shows that lncRNAs have a definitive role in carcinogenesis, invasion and metastasis [15,48]. Our experiments demonstrate that GAS5 expression plays a role in MPM biology.

## Methods

### Tumor specimens

Tumor specimens were obtained for diagnostic purpose before chemotherapy or at the time of resection and were immediately processed as previously described for total RNA extraction using Qiagen RNAeasy^®^ and cDNAs was prepared from 400–500 ng of RNA (Qiagen QuantiTect^®^ Reverse Transcription protocol) [30]. In addition, parts of tumor specimens were fixed in paraformaldehyde for paraffin embedding. Normal pleural tissue was received from ten patients undergoing mesothelioma unrelated thoracic surgery. The study was approved by the Zurich University Hospital ethic committee and a written informed consent was obtained from all patients.

### Cell culture and drug treatment

Primary MPM cultures were established from surgical specimens and maintained with serum and without serum as previously described [25,49]. MPM cells-SPC111, SPC212, ZL34 and ZL55, were established in our laboratory [50] and were maintained in DMEM:F12 (Ham) medium (Sigma-Aldrich, St. Louis, MO, USA) with 15% foetal calf serum (FCS, Invitrogen/GIBCO) and 1% penicillin/streptomycin (Sigma-Aldrich); Mero- 14, Mero-41, Mero-48, Mero-82, Mero-83, Mero-84 and Mero-95 were maintained in DMEM:F10 (Ham) medium (Sigma-Aldrich) with 15% FCS and 1% penicillin/streptomycin; ONE-58, ACC-Meso-1 and ACC-Meso-4 were maintained in RPMI-1640 (Sigma-Aldrich) with 15% FCS and 1% penicillin/streptomycin; MSTO-211H, H2052, H2452, H226, NO36 and H596 cells were maintained in RPMI-1640 with 10% FCS and 1% penicillin/streptomycin. ZL55SPT and SDM103T2 primary cells were grown in serum-free medium [25]. Normal mesothelial cells NP3, SDM77, SDM85, SDM104, SDM58 and SDM71 were cultured as previously described [51]. NP3 cells were also grown in serum-free medium. To induce growth arrest, cells were treated either with Hedgehog signaling inhibitor (HhAntag) [25] or with dual PI3K/mTOR inhibitor (NVP-BEZ235, Novartis, Switzerland) [26], as previously described.

### Gene expression

Selected gene expression analysis using MIQE [52] compliant protocols was conducted as previously described [30]. Briefly, cDNA was amplified by the SYBR-Green PCR assay and products were detected on a 7900HT Fast real-Time PCR system (SDS, ABI/Perkin Elmer). Relative mRNA levels were determined by comparing the PCR cycle thresholds between cDNA of a specific gene and histone (ΔCt). The 5′ and 3′ primers for GAS5 - AAGCCATTGGCACACAGGCATTAG and AGAACCATTAAGCTGGTCCAGGCA, glucocorticoid-induced leucine zipper protein (GILZ) [22] – AACAGGCCATGGATCTGGTGAAGA and AGGGTCTTCAACAGGGTGTTCTCA, serum/glucocorticoid-regulated kinase 1 (SGK1) - CTATGCTGCTGAAATAGC and GTCCGAAGTCAGTAAGG, respectively. RNA extraction and cDNA preparation from cell cultures was achieved as detailed for tumor samples. The heatmap of expression level of GAS5, mesothelin, calretinin and podoplanin was produced as previously described [30,49].

### Plasmid constructs

To investigate the role of GAS5 on cell growth we have generated a system, which allows inducing the silencing of GAS5 by the addition of doxycycline. The shRNA sequence against GAS5 is based on published results [22]. The template for shRNA expression has been obtained by annealing oligonucleotides (GATCCCC *CTTGCCTGGACCAGCTTAA* TTCAAGAGA *TTAAGCTGGTCCAGGCAAG* TTTTTA and AGCTTAAAAA *CTTGCCTGGACCAGCTTAA* TCTCTTGAA *TTAAGCTGGTCCAGGCAAG* GGG). The sense and antisense strands of the 19-nucleotide (nt) which targets nucleotides 199 to 217 of the human GAS5 sequence (GenBank accession number NR_002578) are indicated in italics and are separated by a 9-nt loop sequence (TTCAAGAGA). For the negative control a scrambled sequence was used [22,53]. These 60-base long oligonucleotides containing the coding and complementary sequence of these components, together with BglII and HindIII restriction site sequences at the 5′ and 3′ ends respectively, have been annealed and ligated into pSUPERIOR. PURO vector digested with BglII and HindIII restriction sites. The construct has been checked by DNA sequencing and was deposited in Addgene repository (46370, pSuperior-sh-GAS5).

Stable doxycycline-inducible shGAS5 and sh control clones have been generated by co-transfection of the inducible shGAS5 or control constructs with the plasmid pcDNA6/TR (Invitrogen), encoding high levels of the tetracycline repressor, in human MPM ZL55SPT cells grown in the absence of serum using Lipofectamine 2000 (Invitrogen). After selection with puromycin (0.4 μg/ml) and blasticidin (2.5 μg/ml), stable clones have been isolated. Optimal dose and time for doxycycline to induce silencing of GAS5 was obtained as 0.1 and 0.5 μg/ml for 72 hrs (refreshing doxycycline after 48 hrs). Under these conditions no cell growth alteration, assessed by MTT cell viability assay, was observed in parental ZL55SPT cells.

To investigate the control of GAS5 transcription, 771 bp upstream GAS5 transcription starting site was amplified using the following primers with NheI and HindIII restriction sites:

5′- ACGTGCTAGCTCAGGTGAGAACTAGGAAGG -3′ and

5′- ACGTAAGCTTAAGACAGTATGGTGCCTGGG -3′ and subcloned into pGL3 luciferase reporter (pGL3-B-pGAS5, Addgene 46371).

### RNA interference by siRNA

For down-regulation of *GAS5* with small interfering RNAs (siRNA), ZL55SPT cells were transfected with 25 nM Qiagen siRNAs targeting GAS5: siGAS5_1 FlexiTube siRNA (targeting exon 11), siGAS5_2 FlexiTube siRNA (targeting exon 3), siGAS5_4 FlexiTube siRNA (targeting exons 10–11) or control non targeting (NT) siRNA (Thermo Scientific Dharmacon), according to the manufacturer’s reverse transfection protocol as previously described [25]. Cells were then plated at 7000 cells/cm^2^ and RNA was extracted after 72 h.

### GAS5 promoter reporter assays

To confirm upregulation of GAS5 transcription upon treatment inducing growth arrest dual luciferase assay was used. Briefly, pGL3-B-pGAS5 was transfected together with Renilla Luciferase (50:1) in cells seeded in 12 wells (40’000-100’000 cells/well) as previously described [25]. After 24 h, transfected cells were either treated with HhAntag, NVP-BEZ235 or vehicle for 24 h then were lysed and analyzed using Dual-Luciferase reporter assay system according to manufacturer instruction (Promega, Madison, Wi, USA).

### Analysis of GAS5 splice variants

To identify which GAS5 splice variant is expressed in cell lines that were used in functional assays, we designed three primers sets. PCR reaction was carried out with the following conditions: initial denaturation at 94°C 5 min, then 35 cycles at 95°C 1 min, annealing at 55°C 1 min; and extension at 72°C 2 min. Primer sequences were: GAS5-Ex-4-8-F,- 5′-GTCCTAAAGAGCAAGCCTAACT-3′ and GAS5-Ex-4-8-R,- 5′-TAGTCAAGCCGACTCTCCATA-3′, GAS5-Ex-6-12-F,- 5′-TAATGGTTCTGCTCCTGGTAAC-3′ and GAS5-Ex-4-8-R,- 5′-CAAAGGCCACTGCACTCTA-3′, GAS5-SV-586-F, 5′- CCCAAGGAAGGATGAGAATAGC -3′ and GAS5-SV-586-R, 5′- CTCTTTAGGACCTGGGAAGAAAC -3′. Amplified PCR products were analyzed on 3% agarose gel, then, they were excised from gels, purified (Qiagen Qiaquick^®^ Gel Extraction Kit) and sequenced (Microsynth, Balgach, Switzerland). PCR bands were quantitated with densitometry using Image J software (Version 1.42q, USA).

### Flow cytometric analysis

The analysis of quiescent (G_0_) vs proliferative state (G_1_ or S/G_2_/M) of cells was performed using Attune flow cytometer (Applied Biosystems) and analyzed with the Attune cytometric software v1.2.5 (Applied Biosystems) accordingly to a previously described method [54]. Briefly, cells were fixed with 70% ethanol and stained with FITC-conjugated anti-Ki-67 (clone B56, BD Pharmingen) monoclonal antibody, accordingly to manufacturer instructions. DNA was stained with DAPI (1 μg/ml) after 15 min digestion with RNase A (10 μg/ml).

### EdU incorporation assay

Cell cycle length was measured by Click iT™ EdU cell proliferation assay kit (Molecular Probes, Invitrogen). The EdU (5-ethynyl-2′-deoxyuridine) is a nucleoside analog of thymidine that is incorporated into DNA only during DNA synthesis allowing the visualization of newly synthesized DNA [29]. To perform the assay shGAS5 cells were plated on 4 well chambered slides and incubated at 37°C for overnight to 24 h. The cells were treated with doxycycline for 72 hours with drug refreshment at 48 hrs. At time intervals of 0, 6, 12 and 24 h, cells were treated with 10 μM EdU and incubated at 37°C to ensure capture of the majority of proliferating cells. Following EdU addition, cells were fixed with ice cold 100% methanol for 20 min and permeabilised with ice cold acetone and methanol (50:50) for 20 min followed by 0.2% triton X 100 for 10 min at room temperature. Incorporation of EdU was observed by incubating fixed cells with 2% BSA in PBS for 30 minutes and Alexa fluor 488 for a further 30 minutes under Cu(I)-catalyzed click reaction conditions, as described by the manufacturer. Cells were washed with PBS, and mounted with prolong GOLD anti-fade agent with DAPI (Invitrogen) and visualized under fluorescence microscopy.

### RNA *in-situ* hybridization in MPM cells

To localize GAS5 lncRNA in MPM cells we performed RNA ISH by using QuantiGene^®^ ViewRNA cell assay kit (Panomics Srl, Vignate-Milano, Italy). The oligonucleotide probe was designed commercially using the human GAS5 sequence (accession number NM_002578). ShGAS5 cells in 4-well chamber slides was treated at different concentrations of HhAntag for 48 hours and permeabilized with working detergent solution, and digested with protease at 1:8000 in PBS. The cells were hybridized for 3 hours at 40°C with a cocktail of custom-designed QuantiGene ViewRNA probes against human GAS5 (type 6 probe), RNU2-1 (type 1 probe) and GAPDH (type 4 probe). Unhybridized probes were flushed out with wash buffer and the hybridized probes were amplified with pre-amp hybridization for 1 hour at 40°C, followed by amp hybridization for 1 hour at 40°C. Label Probes (LP) targeting the GAS5, RNU2-1 and GAPDH probe types were added for 30mins at 40°C. Cells were washed with wash buffer and slides were mounted with prolong GOLD anti-fade agent with DAPI and stored at 4°C. The cells were imaged under CLSM Leica SP5 Resonant APD with confocal point-scanning and real optical section (Centre for Microscopy and Image analysis, UZH, Irchel). Twenty five Z stack steps per 0.5 μm at 40 × magnification imaging have been performed for each group with zoom factor fixed as 2. The number of GAS5 lncRNA transcripts inside the nucleus as well in single cell was quantified from z-stacked images of at least 100 randomly selected cells on different fields for each condition using the Spot detection module of Imaris 7.6.1 image analysis software (Bitplane) with fixed spot size and adjusted threshold. Intensity of fluorescent spots of GAS5 lncRNA in both cell and nuclei was measured by filtering the spots with red channel (RNU2-1) and this quantifies the spots which are present in the nucleus of a cell. From the analysed images the total number of fluorescent spots per cell or nucleus was calculated with mean intensity of spots.

### RNA *in-situ* hybridization in MPM tissue

Tissue sections of MPM patients were processed for RNA-ISH by using QuantiGene^®^ ViewRNA ISH tissue assay kit (Panomics Srl, Vignate-Milano, Italy). Briefly, 3- micron sections were deparaffinized, boiled in pre-treatment solution (Affymetrix, Santa Clara, CA) for 5 minutes and digested with proteinase K for 10 minutes. Sections were hybridized for 3 hours at 40°C with custom designed QuantiGene ViewRNA probes against human GAS5 (type 1 probe) and no probe as a control. After the probe hybridization the slides were stored overnight in supplied 1X storage buffer. Hybridized probes were then amplified the following day as per protocol from Affymetrix using PreAmp and Amp molecules. Multiple Label Probe oligonucleotides conjugated to alkaline phosphatase (LP-AP Type 1) were then added and Fast Red Substrate was used to produce signal (red dots). Slides were then counterstained with Hematoxylin. Slides were scanned in Zeiss Mirax Midi Slide Scanner equipped with fluorescence scanner (Centre for Microscopy and Image analysis, UZH, Irchel) and tissue sections were analysed by using Pannoromic viewer 1.15.2 (3DHISTECH, Budapest, Hungary).

### Immunohistochemistry

Deparaffinized sections were subjected to antigen retrieval using Tris/EDTA (pH9.0) or sodium citrate (pH 6.0) buffer. Following quenching in 0.3% H_2_O_2_ (20 min) and permeabilization in 0.05% Saponin (5 min), blocking was performed in 2% bovine serum albumin in PBS with 1% horse serum (20 min; Vector Laboratories) at room temperature. Sections were incubated with primary antibodies (D2-40, 1:50; DAKO M3619, Baar, Switzerland and Ki-67, 1:50; Abcam ab15580, Abcam, Cambridge, UK) overnight at 4°C. Negative controls were incubated with secondary biotinylated antibody only (Vectastain^®^ Elite^®^ ABC Kit; Vector Laboratories, Servion, Switzerland). Sections were washed with PBS and incubated with secondary biotinylated antibody for 45 min at room temperature. Staining was visualized using 3,3′-diaminobenzidine tetrahydrochloride (Sigma–Aldrich), counterstained with Vector^®^ hematoxilyn QS (Vector Laboratories) and analyzed using a Zeiss Mirax Midi Slide Scanner and image acquisition with a 3 CCD color camera and Pannoromic viewer (3DHISTECH, Budapest, Hungary).

### Statistical analysis

Data are expressed as mean ± standard deviation of multiple experiments. Statistical analysis was performed using student *t*-test and Mann–Whitney tests and cell cycle length was analyzed by linear regression and regression slopes were evaluated by StatView 5.0.1 (SAS institute). The comparison of GAS5 expression in PDPN low versus PDPN high samples was performed by dichotomizing the samples according to PDPN median relative expression value. All statistical analysis was performed in GraphPad Prism v5.03. Differences were considered statistically significant at *p* < 0.05.

## Competing interests

The authors declared that they have no competing interests.

## Authors’ contributions

AR, JKR and EFB carried out most of the experiments and participated in the interpretation of the data. GZ carried out the PCR analysis and RNA extraction from the tumors. IO provided clinical samples. BV was involved in the interpretation of the analysis of clinical samples. AR and EFB drafted the manuscript. RS, JKR and NE were involved in revising critically the ms. All authors read and approved the final manuscript.

## Supplementary Material

Additional file 1: Figure S1 Profile of GAS5 lncRNA in different MPM cell line. GAS5 lncRNA expression was analysed by qRT-PCR in 22 MPM cell lines and in 7 normal mesothelial cell lines. Expression of GAS5 was normalized to internal control histones relative to the mean expression of GAS5 in normal mesothelial cells in culture according to –∆∆Ct method. Click here for file

Additional file 2: Table S1 List of GAS5 alternative splice variants. Click here for file

Additional file 3: Table S2 List of GAS5 alternative splice variants detectable by RT-PCR primer sets. Click here for file

Additional file 4: Figure S2 Different GAS5 alternative splice variants are identified in MPM. A. Schematic representation of 3 alternative splice variants of GAS5 expressed in MPM. Gray rectangles represent GAS5 exons, lines represent introns and white rectangle snoRNA. Arrows show the location of primer sets. B, C and D. Semi-quantitative RT-PCR of transcripts for GAS5 differentially expressed in the MPM primary cells and cell lines selected for functional assays. The arrows point to the RT-PCR amplicon corresponding to the different products that can be obtained (207 bp and 168 bp with exons 4 to 8 primer set; 281 bp and 242 bp with exon 6–12 primer set; 233 bp with exon 3 together with SNORD76 (OTTHUMT00000090586, SV-586) . E. GAPDH was amplified from the cDNA and was used for relative quantification. Click here for file

Additional file 5: Figure S3 Cell cycle distribution of ZL55SPT cells upon HhAntag induced growth arrest. HhAntag induced growth arrest accumulates more cells at G_0_ state. Values are shown as mean ± SD from three independent experiments. *; p < 0.05 compared to control. Click here for file

Additional file 6: Figure S4 NVP-BEZ-235 increases GAS5 expression in MPM primary cells ZL55SPT and SDM103T2 grown without serum in 3% oxygen. Values are expressed as mean ± SD from three independent experiments. Click here for file

Additional file 7: Figure S5 Silencing of GAS5 with siRNA increases glucocorticoid responsive genes. A. GAS5 lncRNA silencing increases the expression of glucocorticoid responsive genes GILZ (B) and SGK1 (C). Values are normalized with histones and shown as mean ± SD from four independent experiments. *; p < 0.05. Click here for file

Additional file 8: Figure S6 GAS5 expression in MPM. A. GAS5 expression is higher in MPM tumor samples compared to non-tumoral tissue, *p < 0.0001 expression, Mann–Whitney test. B. Upper panel shows fluorescence *in situ* hybridization of control and GAS5 and immunostaining of Ki-67 (ab15580, Abcam) and lower panel shows the immunostaining of control and podoplanin (D2-40, DAKOCytomation) and hematoxylin and eosin stained tumor of another representative patient. Scale bar: 50 μm. Click here for file

Additional file 9: Figure S7 *GAS5* promoter in Chromosome 1 region 1q25.1. A screenshot of the UCSC Genome Browser shows *GAS5* sharing its promoter with *ZBTB37*. Click here for file
