# Supplementary figures and images for: GAS5 long non-coding RNA in malignant pleural mesothelioma
Source: Mol Cancer. 2014 May 23;13:119. doi: 10.1186/1476-4598-13-119 (PMC4039656; doi:10.1186/1476-4598-13-119)

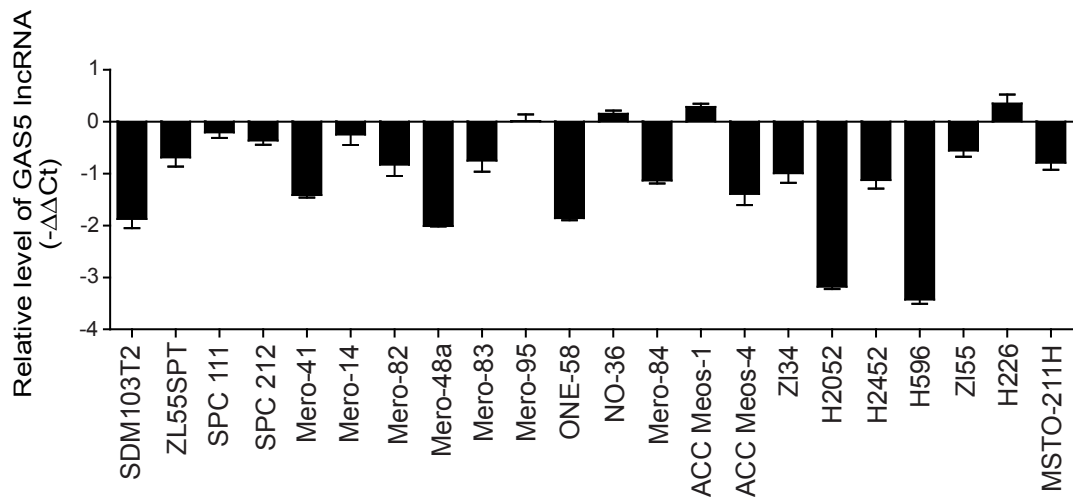

Figure S1

Supplement: Additional file 1: Figure S1 — Profile of GAS5 lncRNA in different MPM cell line. GAS5 lncRNA expression was analysed by qRT-PCR in 22 MPM cell lines and in 7 normal mesothelial cell lines. Expression of GAS5 was normalized to internal control histones relative to the mean expression of GAS5 in normal mesothelial cells in culture according to –∆∆Ct method. [file 1476-4598-13-119-S1.pdf]

A)

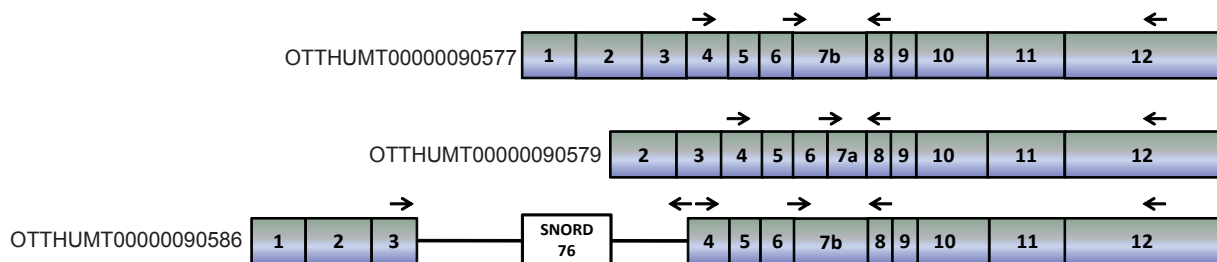

B)

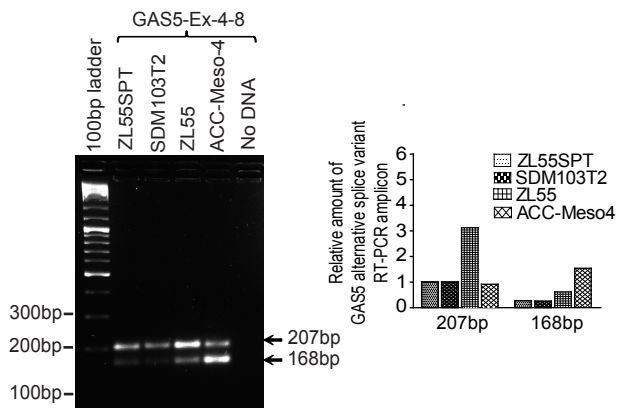

C)

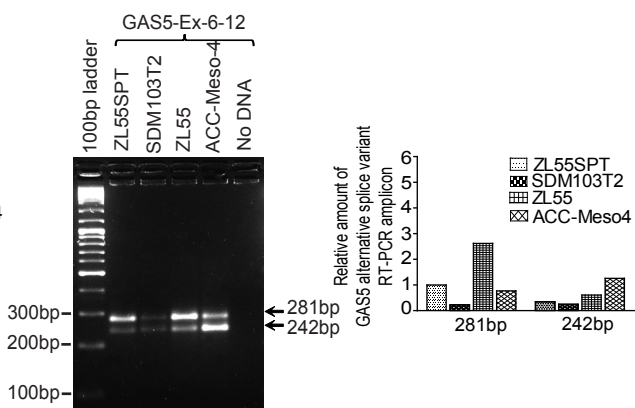

D)

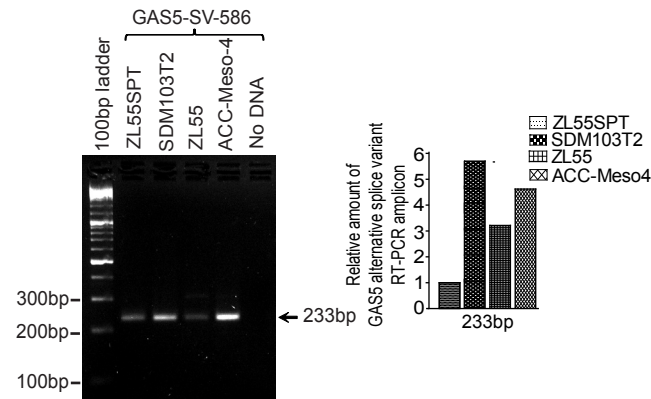

E)

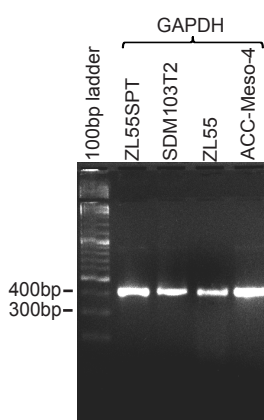

Figure S2

Supplement: Additional file 4: Figure S2 — Different GAS5 alternative splice variants are identified in MPM. A. Schematic representation of 3 alternative splice variants of GAS5 expressed in MPM. Gray rectangles represent GAS5 exons, lines represent introns and white rectangle snoRNA. Arrows show the location of primer sets. B, C and D. Semi-quantitative RT-PCR of transcripts for GAS5 differentially expressed in the MPM primary cells and cell lines selected for functional assays. The arrows point to the RT-PCR amplicon corresponding to the different products that can be obtained (207 bp and 168 bp with exons 4 to 8 primer set; 281 bp and 242 bp with exon 6–12 primer set; 233 bp with exon 3 together with SNORD76 (OTTHUMT00000090586, SV-586) . E. GAPDH was amplified from the cDNA and was used for relative quantification. [file 1476-4598-13-119-S4.pdf]

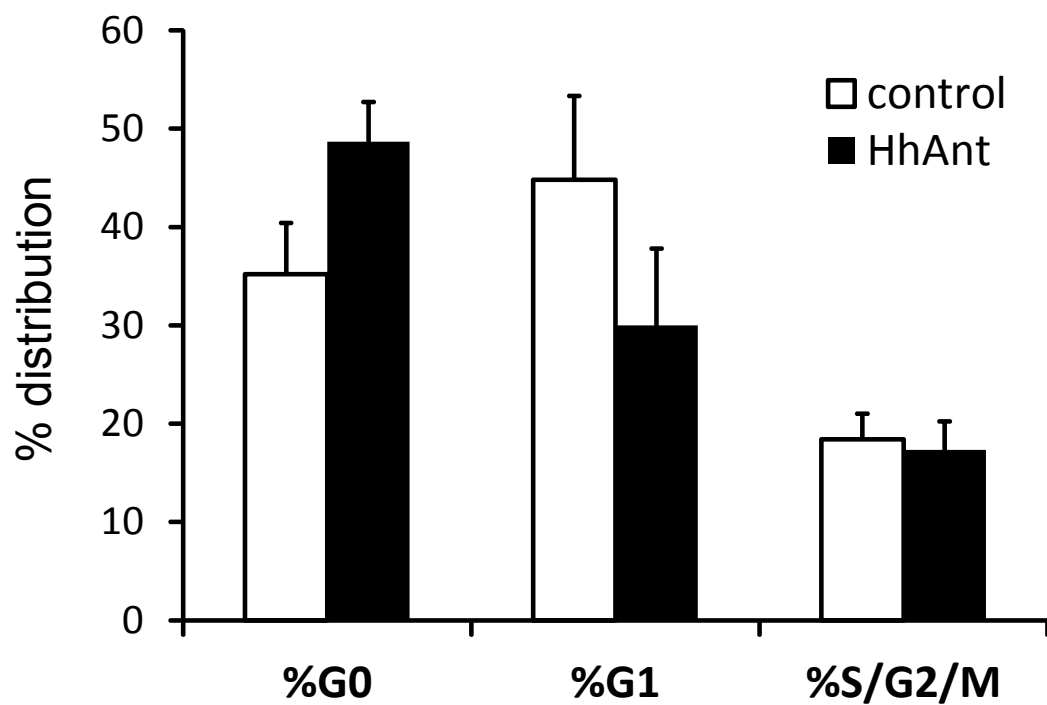

Figure S3

Supplement: Additional file 5: Figure S3 — Cell cycle distribution of ZL55SPT cells upon HhAntag induced growth arrest. HhAntag induced growth arrest accumulates more cells at G0 state. Values are shown as mean ± SD from three independent experiments. *; p < 0.05 compared to control. [file 1476-4598-13-119-S5.pdf]

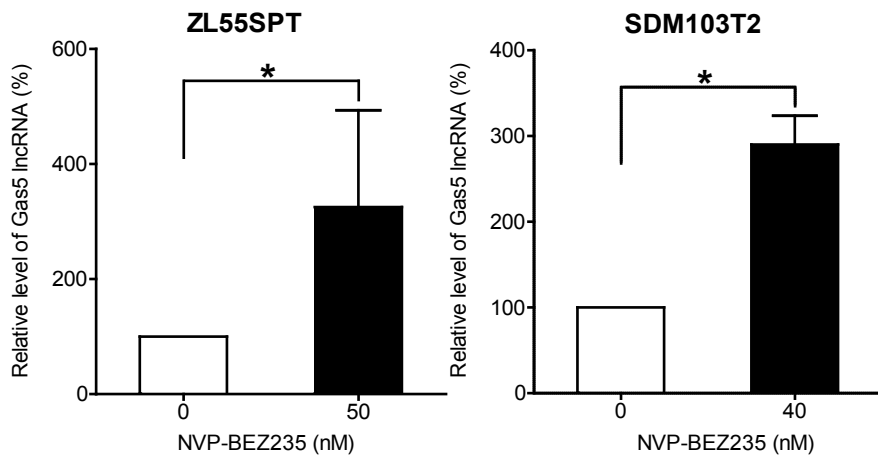

Figure S4

Supplement: Additional file 6: Figure S4 — NVP-BEZ-235 increases GAS5 expression in MPM primary cells ZL55SPT and SDM103T2 grown without serum in 3% oxygen. Values are expressed as mean ± SD from three independent experiments. [file 1476-4598-13-119-S6.pdf]

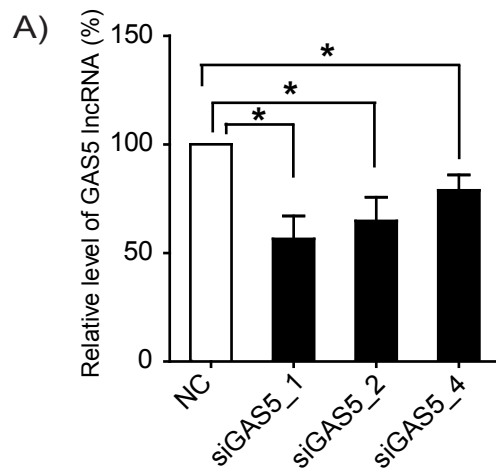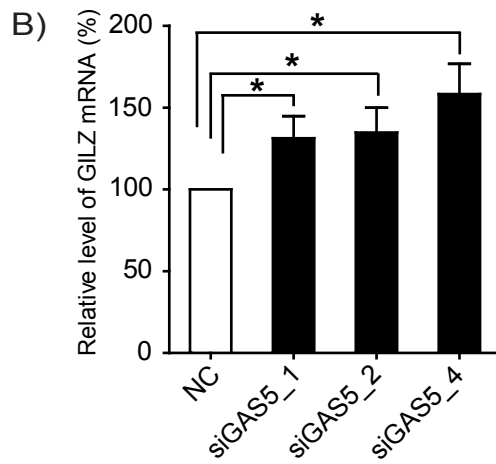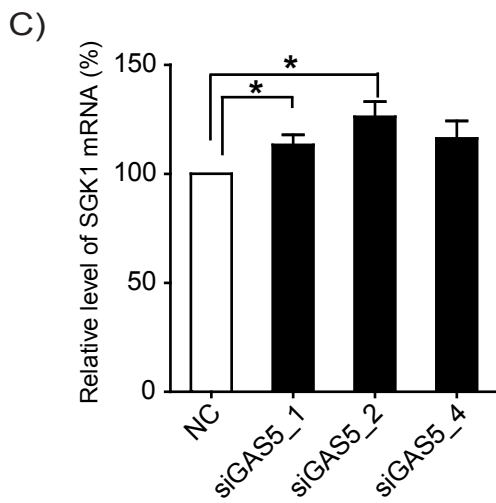

Figure S5

Supplement: Additional file 7: Figure S5 — Silencing of GAS5 with siRNA increases glucocorticoid responsive genes. A. GAS5 lncRNA silencing increases the expression of glucocorticoid responsive genes GILZ (B) and SGK1 (C). Values are normalized with histones and shown as mean ± SD from four independent experiments. *; p < 0.05. [file 1476-4598-13-119-S7.pdf]

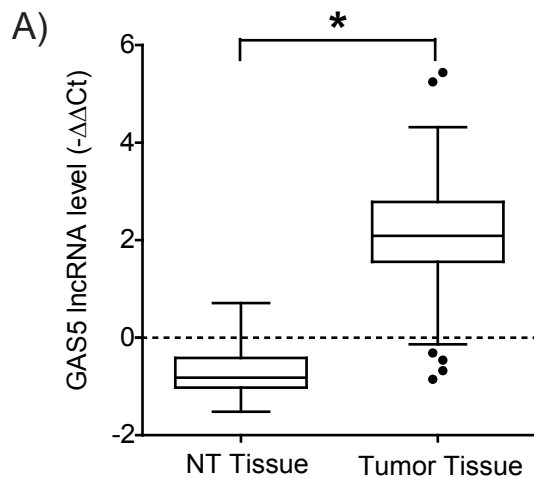

B)

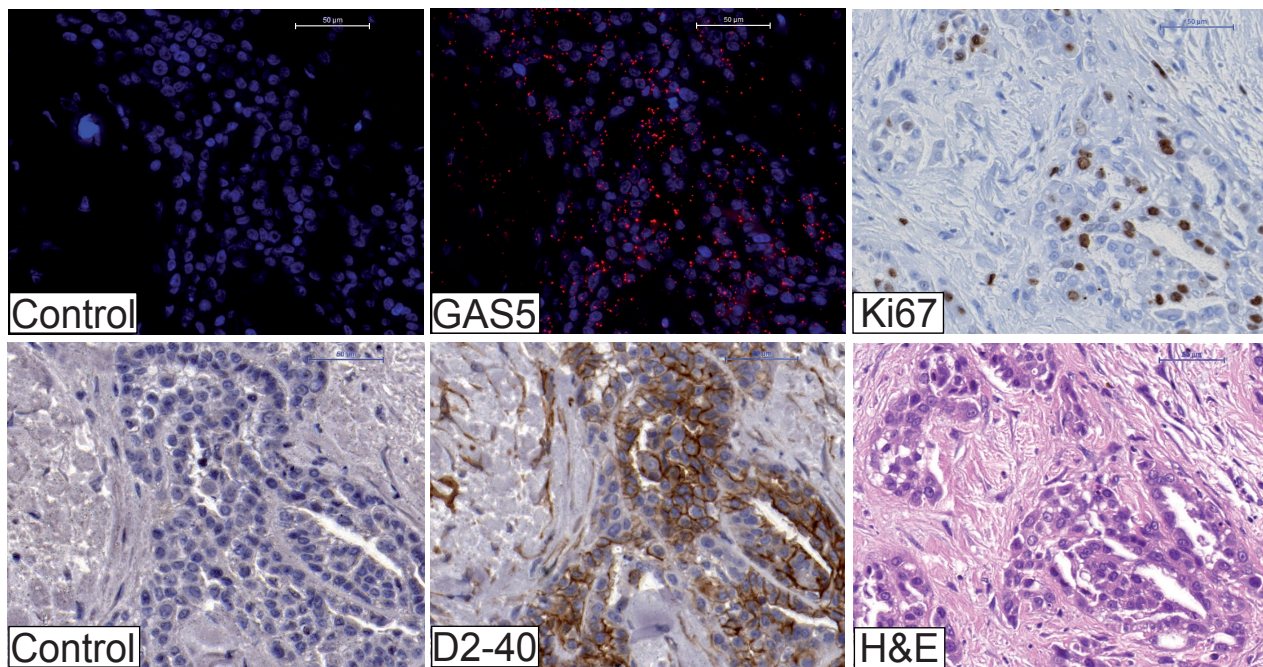

Figure S6

Supplement: Additional file 8: Figure S6 — GAS5 expression in MPM. A. GAS5 expression is higher in MPM tumor samples compared to non-tumoral tissue, *p < 0.0001 expression, Mann–Whitney test. B. Upper panel shows fluorescence in situ hybridization of control and GAS5 and immunostaining of Ki-67 (ab15580, Abcam) and lower panel shows the immunostaining of control and podoplanin (D2-40, DAKOCytomation) and hematoxylin and eosin stained tumor of another representative patient. Scale bar: 50 μm. [file 1476-4598-13-119-S8.pdf]
