# Supplementary material for: GAS5 long non-coding RNA in malignant pleural mesothelioma
Source: Mol Cancer. 2014 May 23;13:119. doi: 10.1186/1476-4598-13-119 (PMC4039656; doi:10.1186/1476-4598-13-119)
Supplement: Additional file 2: Table S1 — List of GAS5 alternative splice variants. [file 1476-4598-13-119-S2.pdf]

Supplementary Table 1 – Different splice variants of *GAS5* gene from Vega genome browser

http://vega.sanger.ac.uk/Homo_sapiens/Gene/Summary?g=OTTHUMG00000037216;r=1:173833038-173838020;db=vega_update

Gene: GAS5 OTTHUMG00000037216

Description growth arrest-specific 5 (non-protein coding)

Location Chromosome 1: 173,833,038-173,838,020 reverse strand.

Transcripts This gene has 29 transcripts (splice variants) Hide transcript table

Name	Transcript ID	Length (bp)	Protein ID	Length (aa)	Biotype	CCDS
GAS5-016	OTTHUMT00000090592	841	No protein product	-	LincRNA	-
GAS5-015	OTTHUMT00000090591	796	No protein product	-	LincRNA	-
GAS5-027	OTTHUMT00000090603	723	No protein product	-	LincRNA	-
GAS5-014	OTTHUMT00000090590	688	No protein product	-	LincRNA	-
GAS5-001	OTTHUMT00000090577	632	No protein product	-	LincRNA	-
GAS5-003	OTTHUMT00000090579	565	No protein product	-	LincRNA	-
GAS5-008	OTTHUMT00000090584	542	No protein product	-	LincRNA	-
GAS5-013	OTTHUMT00000090589	483	No protein product	-	LincRNA	-
GAS5-028	OTTHUMT00000090604	413	No protein product	-	LincRNA	-
GAS5-020	OTTHUMT00000090596	242	No protein product	-	LincRNA	-
GAS5-002	OTTHUMT00000090578	1698	No protein product	-	Retained intron	-
GAS5-018	OTTHUMT00000090594	1114	No protein product	-	Retained intron	-
GAS5-006	OTTHUMT00000090582	1060	No protein product	-	Retained intron	-
GAS5-022	OTTHUMT00000090598	979	No protein product	-	Retained intron	-
GAS5-011	OTTHUMT00000090587	897	No protein product	-	Retained intron	-
GAS5-004	OTTHUMT00000090580	822	No protein product	-	Retained intron	-
GAS5-012	OTTHUMT00000090588	799	No protein product	-	Retained intron	-
GAS5-017	OTTHUMT00000090593	772	No protein product	-	Retained intron	-
GAS5-019	OTTHUMT00000090595	745	No protein product	-	Retained intron	-
GAS5-029	OTTHUMT00000090605	723	No protein product	-	Retained intron	-
GAS5-010	OTTHUMT00000090586	712	No protein product	-	Retained intron	-
GAS5-023	OTTHUMT00000090599	643	No protein product	-	Retained intron	-
GAS5-009	OTTHUMT00000090585	632	No protein product	-	Retained intron	-
GAS5-026	OTTHUMT00000090602	621	No protein product	-	Retained intron	-
GAS5-007	OTTHUMT00000090583	583	No protein product	-	Retained intron	-
GAS5-024	OTTHUMT00000090600	575	No protein product	-	Retained intron	-
GAS5-021	OTTHUMT00000090597	497	No protein product	-	Retained intron	-
GAS5-005	OTTHUMT00000090581	469	No protein product	-	Retained intron	-
GAS5-025	OTTHUMT00000090601	424	No protein product	-	Retained intron	-

Summary

Synonyms NCRNA00030, SNHG2 [To view all genes linked to the name click here.]

Gene type Known Processed transcript [Definition]

Author This gene was annotated by Havana <vega@sanger.ac.uk>

Version & date Version 3, last modified on 23/10/2013 (Created on 04/05/2004)

Remarks ncRNA host
