# Supplementary material for: GAS5 long non-coding RNA in malignant pleural mesothelioma
Source: Mol Cancer. 2014 May 23;13:119. doi: 10.1186/1476-4598-13-119 (PMC4039656; doi:10.1186/1476-4598-13-119)
Supplement: Additional file 3: Table S2 — List of GAS5 alternative splice variants detectable by RT-PCR primer sets. [file 1476-4598-13-119-S3.pdf]

Supplementary Table 2 - List of GAS5 alternative splice variants detectable by RT-PCR primer sets. Sequence of primers was matched with human cDNA database by using BLASTN search tool with exact match sensitivity (Vega Genome Browser 54). Transcripts ID that were common to both primers sets are in bold.

**Vega Genome Browser 54 Transcript ID**	
**GAS5-Ex-4-8**	**GAS5-Ex-6-12**
**OTTHUMT00000090577**	**OTTHUMT00000090577**
**OTTHUMT00000090578**	**OTTHUMT00000090578**
**OTTHUMT00000090579**	**OTTHUMT00000090579**
**OTTHUMT00000090580**	**OTTHUMT00000090580**
OTTHUMT00000090583	OTTHUMT00000090584
OTTHUMT00000090585	**OTTHUMT00000090586**
**OTTHUMT00000090586**	**OTTHUMT00000090590**
OTTHUMT00000090588	**OTTHUMT00000090593**
OTTHUMT00000090589	OTTHUMT00000090595
**OTTHUMT00000090590**	OTTHUMT00000090598
**OTTHUMT00000090593**	OTTHUMT00000090603
OTTHUMT00000090594	OTTHUMT00000090604
OTTHUMT00000090595	OTTHUMT00000090605
OTTHUMT00000090597	
