# Supplementary material for: GAS5 long non-coding RNA in malignant pleural mesothelioma
Source: Mol Cancer. 2014 May 23;13:119. doi: 10.1186/1476-4598-13-119 (PMC4039656; doi:10.1186/1476-4598-13-119)
Supplement: Additional file 9: Figure S7 — GAS5 promoter in Chromosome 1 region 1q25.1. A screenshot of the UCSC Genome Browser shows GAS5 sharing its promoter with ZBTB37. [file 1476-4598-13-119-S9.pdf]

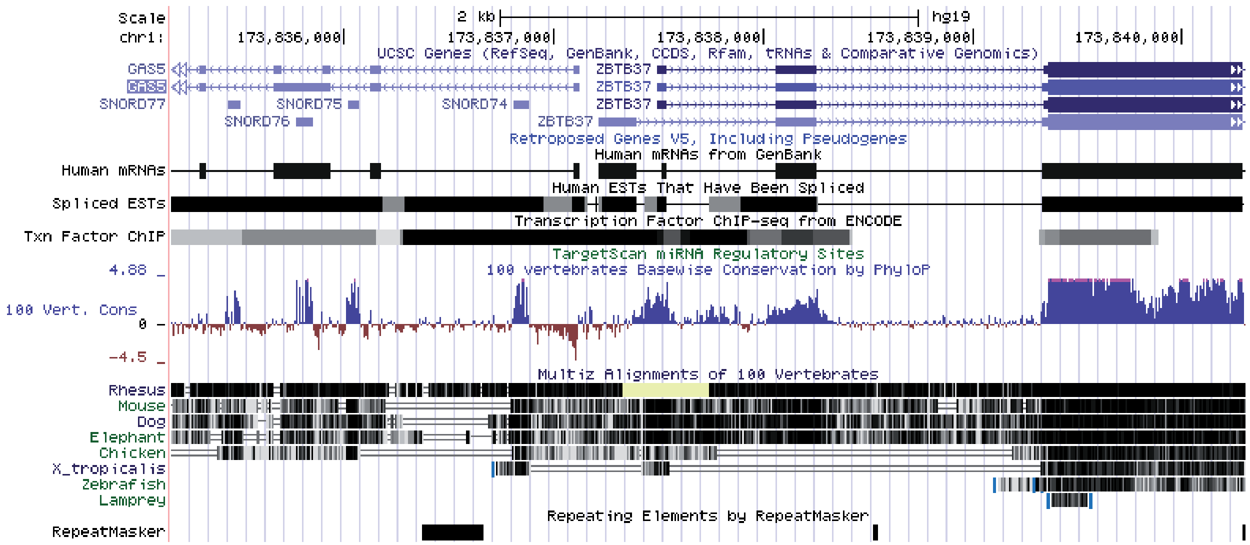
Scale
chr1:
2 kb
hg19
173,836,000
173,837,000
173,838,000
173,839,000
173,840,000
UCSC Genes (RefSeq, GenBank, CCDS, Rfam, tRNAs & Comparative Genomics)
GAS5
GAS5
SNORD77
SNORD75
SNORD74
SNORD76
ZBTB37
ZBTB37
ZBTB37
ZBTB37
Retroposed Genes V5, Including Pseudogenes
Human mRNAs from GenBank
Human mRNAs
Human ESTs That Have Been Spliced
Spliced ESTs
Transcription Factor ChIP-seq from ENCODE
Txn Factor ChIP
TargetScan miRNA Regulatory Sites
100 vertebrates Basewise Conservation by PhyloP
4.88
100 Vert. Cons
0
-4.5
Multiz Alignments of 100 Vertebrates
Rhesus
Mouse
Dog
Elephant
Chicken
X_tropicalis
Zebrafish
Lamprey
Repeating Elements by RepeatMasker
RepeatMasker

Figure S7
